# Cost-effectiveness analysis of mepolizumab among patients with severe asthma from the Chinese societal perspective

**DOI:** 10.1371/journal.pone.0348955

**Published:** 2026-05-13

**Authors:** Chaohao Shi, Yang Wang, Chongqing Tan, Xiaohui Zeng, Qiao Liu

**Affiliations:** 1 Department of Pharmacy, The Second Xiangya Hospital of Central South University, Changsha, China; 2 Institute of Clinical Pharmacy, Central South University, Changsha, China; 3 Department of Pathology, The Second Xiangya Hospital, Central South University, Changsha, China; 4 Hunan Clinical Medical Research Center for Cancer Pathogenic Genes Testing and Diagnosis, Changsha, China; 5 Department of Nuclear Medicine, The Second Xiangya Hospital of Central South University, Changsha, China; Hospital Infantil de México Federico Gomez: Hospital Infantil de Mexico Federico Gomez, MEXICO

## Abstract

**Background:**

Patients with severe asthma, particularly those with an eosinophilic phenotype, face a limited array of treatment options. The Mepolizumab Efficacy in Patients with Severe Asthma (MENSA) trial (clinicaltrials.gov NCT03562195) has underscored the potential therapeutic benefits of mepolizumab as an adjunctive treatment for this patient group. However, the high cost of mepolizumab therapy presents significant economic challenges that may impede its widespread adoption in China. This study aims to assess the cost-effectiveness of mepolizumab from a societal perspective within the Chinese population suffering from severe asthma.

**Methods:**

A Markov model spanning a lifetime horizon and employing a 2-week cycle was constructed. The model incorporated clinically significant exacerbations (CSEs) rates from the MENSA trial, along with asthma mortality rates, costs, and health state utilities, sourced from a broad spectrum of literature and national databases. Both costs and effectiveness outcomes were discounted annually at 5%. Incremental cost-effectiveness ratios (ICERs) were calculated as the primary model output and compared to a willingness-to-pay (WTP) threshold range of $15,217 to $38,042 per quality-adjusted life-years (QALY). The model’s robustness was evaluated through sensitivity analyses.

**Results:**

The base-case analysis indicated an ICER of $848.79 per QALY for mepolizumab, fall below the lower limit of the predefined WTP threshold. Subgroup analyses confirmed this finding across different subpopulations. Deterministic sensitivity analyses showed that mepolizumab’s ICER remained below the lower limit of the predefined WTP threshold even under significant parameter variations. Probabilistic sensitivity analyses further demonstrated mepolizumab’s cost-effectiveness advantage over placebo.

**Conclusion:**

Our study concluded that mepolizumab is a cost-effectiveness treatment option for severe asthma in China, applicable across various patient subgroups. This finding is crucial for healthcare decision-makers in China, where balancing cost and health outcomes is essential due to limited resources.

## Introduction

Severe asthma, as definded by both Chinese asthma guidelines and the Global Initiative for Asthma guidelines, requiers the most intensive treatment, including high-dose inhaled corticosteroids paired with a second controller medication or oral corticosteroids (OCS) to manage symptoms. It is a condition that often remains uncontrolled despite these aggressive treatments [1,2]. In China, asthma poses a significant public health challenge, impacting 4.2% of the population [3], with about 6% of these cases classified as severe [4], aligning with global prevalence rates (5–10%) [5]. Individuals with severe asthma commonly face recurrent exacerbations, suboptimal symptom control, diminished quality of life, and lung function deficits [1]. The Global Burden of Disease study ranks asthma as the 8th leading cause of disability-adjusted life years in China [6], highlighting its profound impact on public health.

Eosinophilic asthma is a distinct form characterized by eosinophilia in sputum and elevated peripheral blood eosinophils, or increased fractional exhaled nitric oxide levels [7]. While several biologics have been approved globally for severe asthma management [8], in China, only omalizumab is approved for moderate-to-severe allergic asthma, leaving no approved biologics for the eosinophilic subtype [9]. This gap underscores the urgent need for a safe and effective treatment to alleviate the disease burden and enhance therapeutic options for patients with severe asthma in China. Mepolizumab is a humanized monoclonal antibody that specifically targets interleukin (IL)-5, the key cytokine responsible for eosinophil differentiation, activation, and survival [10]. In China, mepolizumab marks a significant milestone as the first biologic therapy approved for eosinophilic-driven conditions, including severe asthma with an eosinophilic phenotype [11]. Its approval was bolstered by multiple Phase III clinical trials, which revealed that mepolizumab significantly decreases exacerbation rates, reduces the need for oral corticosteroid use, and enhances asthma control, lung function, and overall quality of life compared to placebo [12–15].

Despite its promise, the high cost of mepolizumab, priced at $682.02 per 100 mg dose [16], presents a barrier to its widespread use in China. A cost-effectiveness analysis is essential to determine if the benefits of mepolizumab justify its cost. The lack of clinical studies within China has previously hindered such an analysis. The recent MENSA Phase III trial (clinicaltrials.gov identifier NCT03562195) filled this gap by investigating mepolizumab‘s efficacy and safety in a Chinese severe asthma population [17]. Building on this data, we performed an economic evaluation to assess the clinical and economic impacts of mepolizumab use among Chinese patients with severe asthma.

The rationale for this study is rooted in the critical need to assess the cost-effectiveness of mepolizumab in China, where its high cost and lack of alternatives for eosinophilic asthma necessitate an evaluation of its affordability. By utilizing a Markov model to simulate the long-term course of severe asthma and its associated economic and clinical impacts, our analysis addresses this need. The results are pivotal for Chinese healthcare decision-makers, who navigate limited resources while striving to balance cost and health outcomes. By elucidating the cost-effectiveness of mepolizumab across different patient subgroups, this research provides a robust basis for its rational clinical application, potentially enhancing the quality of life for patients with severe asthma and ensuring the optimal use of healthcare resources.

This study uses a societal perspective for cost-effectiveness analysis, including both direct medical costs and indirect costs like productivity loss [18]. In China, this perspective is particularly relevant given patients’ and families’ significant out-of-pocket expenses [19], especially for high-cost and chronic treatments-long-term, intensive care for chronic conditions further increases household financial burden. Considering these social factors enables a comprehensive evaluation of the treatment’s societal economic impact, critical for China’s health policy development.

## Materials and methods

### Analytical overview

This economic evaluation of mepolizumab, grounded in the MENSA trial data, employed a Markov model-a stochastic simulation tool specifically crafted for modeling the progression of chronic diseases. This approach facilitated the assessment of long-term cost-effectiveness by tracking the dynamics of health state transitions across time. The study’s population precisely mirrored the MENSA trial’s cohort, adhering to the same stringent inclusion and exclusion criteria. Participants were individuals diagnosed with eosinophilic severe asthma and persistent airflow obstruction, with an average age of 52.2 years [17]. The original MENSA trial publication provides comprehensive details on the specific criteria used for participant selection.

This study adopted the Chinese societal perspective to account for a broader spectrum of disease-associated costs. The model calculated the overall costs for Chinese patients with severe asthma, considering treatment with or without mepolizumab. It assessed therapeutic effectiveness in terms of quality-adjusted life-years (QALYs) and computed the incremental cost-effectiveness ratio (ICER) for mepolizumab therapy versus placebo. Both costs and QALYs were adjusted using a half-cycle correction and discounted annually at a rate of 5% [20]. All costs were presented in Chinese Yuan (¥) and US dollar ($), with an exchange rate of 1 US dollar to 7.0467 ¥ [21].

The study rigorously adhered to the China Guidelines for Pharmacoeconomic Evaluations (2020 Edition) and the Consolidated Health Economic Evaluation Reporting Standards (CHEERS) to ensure methodological integrity in model design, analysis, and reporting [20,22]. This study is based on the analysis of aggregated data from published sources and does not involve direct human subject interaction or the collection of personal identifiable information. Consequently, it was classified as non-human subjects research and was exempt from ethical review by the Clinical Ethics Committee. As such, an Ethical Approval Number is not applicable to this study.

### Markov model structure

**Fig 1:**
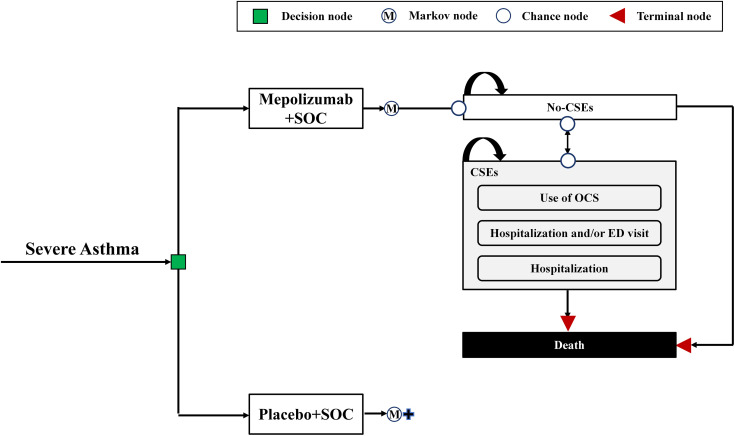
Schematic diagram of the Markov Model structure. SOC, standard of care; CSEs, clinically significant exacerbations; OCS, oral corticosteroid; ED,emergency department.

The Markov model simulated the progression of severe asthma patients across three mutually health states: No-clinically significant exacerbations (No-CSEs), CSEs, and death, as illustrated in Fig 1. Acknowledging severe asthma as an incurable chronic condition, our model was constructed to utilize a full lifetime horizon for analysis, which entails simulating outcomes until the terminal event of death is reached for all participants. Based on previous economic evaluations [23], and considering that typical CSEs usually last less than two weeks, using a shorter cycle better represents the actual clinical dynamics [24].Accordingly, the Markov cycle was set at 2 weeks to reflect the typical recovery period for acute CSEs.

All patients initiated in the No-CSEs health state and were randomly assigned in a 1:1 ratio to receive either mepolizumab 100 mg or placebo via subcutaneous injection every 4 weeks. Patients was assumed to maintain their existing standard-of-care (SOC) maintenance therapy throughout the study period. Dosage and administration for both treatment are provided in S1 Table. Within each Markov cycle, patients faced the risk of transitioning to the CSEs health state due to disease exacerbation, or mortality. The CSEs health state was further subdivided into three substates based on severity and healthcare resource utilization: use of oral corticosteroids, requiring hospitalization and/or emergency department (ED) visit, and requiring hospitalization. Patients within the CSEs state were assumed to receive appropriate disease management in line with the Guidelines for Prevention and Treatment of Bronchial Asthma (2020 Edition) [25].

### Transition probability

#### CSEs rates.

The CSEs events rates were were derived from data of the MENSA trial. CSEs events were identified as episodes of asthma worsening that necessitated systemic corticosteroid use, and/or ED visits, and/or hospitalization. The annual CSE rates were 0.45 and 1.31 in the mepolizumab and placebo arms, respectively. Considering the reversibility between CSE and non-CSE health states and the 2-week Markov cycle adopted in this analysis, these annual rates were converted to biweekly rates, yielding values of 0.01731 (mepolizumab arm) and 0.05038 (placebo arm). In our model, the 52-week efficacy data from the MENSA trial were extrapolated to a lifetime horizon by assuming a constant CSEs incidence rate over the entire simulation period. This assumption was justified by the stable CSEs rate observed across the 52-week trial duration, with no statistically significant temporal trends in CSEs occurrence reported in the trial dataset.

For subgroup analyses, a uniform CSE rate was applied to all subgroups in the placebo arm owing to the lack of subgroup-specific CSE incidence data. Mepolizumab-specific CSEs rates for individual subgroups were then calculated by multiplying this uniform placebo-arm rate by the subgroup-level CSEs rate ratios (RRs).

In addition, three CSEs-specific proportional rates were defined to characterize the intervention pathways following CSEs onset, all of which were extracted from the MENSA trial:

1. Proportion of CSEs events managed with OCS
2. Proportion of CSEs events requiring hospitalisation and/or ED visit
3. Proportion of CSEs events requiring hospitalization

These proportional rates were computed by dividing the number of events corresponding to each intervention type by the total number of CSE episodes, thus reflecting the distribution of post-exacerbation management strategies across all CSE events. Detailed parameters for all CSE rates are provided in S2 Table.

#### Asthma mortality rates.

To calculate age-specific asthma mortality rates, we integrated three distinct datasets in a stepwise manner: First, we extracted 2020 age- and gender-stratified counts of asthma-related deaths from a 30-year retrospective study that quantified the national burden of asthma mortality in China [26]. Second, we sourced age- and gender-specific asthma incidence rates from the China Pulmonary Health Study—a nationwide cross-sectional survey with a representative sample of 57,779 adults spanning 10 provinces, covering diverse socioeconomic contexts and six geographical regions [3].

By combining these two datasets, we derived age- and gender-specific asthma mortality rates for males and females, respectively. Finally, we incorporated 2020 population data from the National Bureau of Statistics [27], weighting the gender-stratified mortality rates by the corresponding gender-specific population sizes to generate overall age-specific asthma mortality rates (gender-aggregated) for the general population. The data sources on asthma mortality rate calculation are detailed in S3 Table.

For patients experiencing CSEs, the age-specific asthma mortality rates were derived from a systematic retrospective, observational study [28]. These annual rates were then converted to bi-weekly rates using a specific formula: $R_{Bi-weekly}=\text{1}-\sqrt[{\text{2}\text{6}}]{(\text{1}-R_{annual}}$ [29]. Parameters for age-specific asthma mortality rates are provided in S4 Table.

### Costs

The model incorporates both direct and indirect costs, with all cost parameters derived from 2024 data. Relevant details are provided in S5 Table.

Direct medical costs encompass expenses for drug acquisition, routine monitoring, CSEs treatment, and adverse events (AEs) management. Drug acquisition costs were calculated using drug unit prices, dosages, and administration frequencies, with unit prices sourced from the latest bid-winning prices available in the National Health Industry Data Platform [30].The bi-weekly monitoring cost and CSEs management cost were derived from previous literature conducted in 2024 [25].

The model also accounts for the costs of managing common on-treatment AEs. The AEs analyzed were derived from the MENSA study, encompassing all on-treatment events with an incidence of ≥5%. These include: asthma, upper respiratory tract infection, nasopharyngitis, bronchitis, allergic rhinitis, productive cough, pneumonia, headache, cough, dizziness, pharyngitis, rhinitis, arthralgia, and oropharyngeal pain [17].The cost of AEs per event is determined based on China’s Diagnosis-Related Groups policy, which establishes a payment standard for diseases classified according to the Diagnosis Classification and Codes (ICD-10) [31]. Specifically, the latest DRG/DIP 2.0 payment data were used in this study, which were obtained from the Aiden Doctor WeChat mini-program that reflects current clinical payment standards across medical institutions in China. For each AE in our model, the corresponding industry average medical fees are determined via the ICD-10 directory, ensuring a standardized approach to cost inclusion (S6 Table). Subsequently, the AEs management cost for each treatment arm is estimated by multiplying the frequency of AEs by the determined cost per AE event.

The indirect costs associated with asthma-induced productivity loss were estimated by calculating the product of missed paid workdays and the average daily wage, with adjustments made according to employment rates. Information on daily wages was sourced from the National Bureau of Statistics [21]. Employment rates for different age groups were estimated using data from the China Population Census Yearbook 2020 [27]. For patients experiencing CSEs, the missed workdays were determined as one day for those who received outpatient prescriptions and 6.4 days for those who required hospitalization [32]. The assumption regarding the extent of productivity loss in terms of presenteeism days among patients with CSEs were informed by the economic burden of asthma in Singapore [33].

### Health state utilities

We obtained the utility value for patients in the No-CSEs health state through a systematic literature review and meta-analysis that synthesized data from 52 relevant studies, assigning it a score of 0.84 [34]. Disutilities linked to CSEs managed with OCS, hospitalization and/or ED visit, and hospitalization were extracted from an earlier economic assessment [23].

To evaluate the impact of on-treatment AEs on health state utilities, we employed a frequency-weighted summation technique. Disutilities for individual AEs were sourced from the Institute for Clinical and Economic Review (S6 Table) [35]. Thereafter, the disutility attributable to AEs within each treatment arm was computed by multiplying the frequency of each AE by its corresponding disutility. Parameters for health state utilities are outlined in S7 Table.

### Statistical analysis

#### Base-case ICERs.

The model utilized TreeAge Pro Healthcare software (version 2022, https://www.treeage.com/) for statistical analysis. The primary outcome was the ICERs comparing mepolizumab therapy to placebo. These ICERs were then assessed against a predetermined willingness-to-pay (WTP) threshold to evaluate the relative cost-effectiveness of mepolizumab.

This study adopted the evidence-based Willingness-to-Pay (WTP) threshold range of 1.2–3.0 × China’s 2023 per-capita GDP ($15,217 to $38,042 per QALY), as recommended by Cai et al. [36]. Based on the value of statistical life, Cai et al. estimated China’s quality-adjusted life year (QALY)-based cost-effectiveness threshold (CET) to be approximately 1.5×per-capita GDP, with a reasonable range of 1.2–3.0×per-capita GDP. Notably, this threshold range has been widely applied in cost-effectiveness analyses of biologic therapies for chronic diseases in the Chinese context [37]. Mepolizumab therapy was deemed cost-effective if the ICER fell below the predetermined WTP threshold, and non-cost-effective if it did not.

#### Subgroup-level ICERs.

Subgroup-level ICERs were additionally examined to delve into the cost-effectiveness outcomes of mepolizumab therapy within specific subgroups. The subgroup-level rate ratios (RRs) of the CSEs rate between mepolizumab therapy and placebo, as detailed in S2 Table, were employed in these analyses. To derive the subgroup-level CSEs rate for the mepolizumab arm, the RRs were multiplied by the CSEs rate in the placebo arm.

#### Sensitivity analysis.

To validate the stability of the model outcomes, a comprehensive set of sensitivity analyses were conducted. The deterministic sensitivity analyses (DSA) examined the isolated effects of over 50 model parameters by adjusting their values within reasonable limits. This encompassed a ± 20% variation for the majority of parameters (consistent with established practices in comparable cost-effectiveness studies of asthma and biologic therapies [38,39]), 95% confidence intervals for RRs, and literature-derived range values for the No-CSEs health state utility.

Additionally, probabilistic sensitivity analyses (PSA) were executed through 10,000 Monte Carlo simulations to assess the combined impact of varying multiple parameters. In the PSA, parameters followed appropriate distributions as proposed by the ISPOR-SMDM Making Modeling Good Research Practices Task Force [40]. The specific ranges for DSA and the distributions for PSA are delineated in S2, S5, and S7 Tables.

### Ethics statements

Not applicable.

## Results

### Base-case ICERs

In treating Chinese patients with severe asthma, mepolizumab resulted in a slight cost increase of $399.17, rising from $30,302.61 to $30,701.78, and a modest enhancement in QALYs by 0.47028, from 13.04657 to 13.51685, compared to placebo. The ICER for mepolizumab over placebo was $848.79 per QALY, below the lower limit of the predefined WTP threshold, which ranged from $15,217 to $38,042 per QALY (S8 Table).

### Subgroup-level ICERs

As presented in S8 Table, mepolizumab demonstrated cost savings and improved survival in specific subgroups, including those aged 65 years or older, with a weight between 60 and 75 kg, a baseline predicted pre-bronchodilator FEV_1_ of 60% or less, three exacerbations in the year before screening, and a blood eosinophil count below 150 at screening, resulting in its dominance in these populations. The relatively lower total costs in these subgroups could be explained by the offsetting effects from reduced indirect costs, including fewer AEs, lower CSEs-related expenses, and less productivity loss.

For other subgroups, mepolizumab was associated with higher costs but also superior survival outcomes, with ICERs ranging from $ 257.30 to $8,490.85 per QALY, all of which remained consistently beneath the lower threshold of the established WTP range. The generally higher costs in most mepolizumab treated subgroups were mainly driven by the additional drug cost of mepolizumab itself..

### Sensitivity analysis

**Fig 2:**
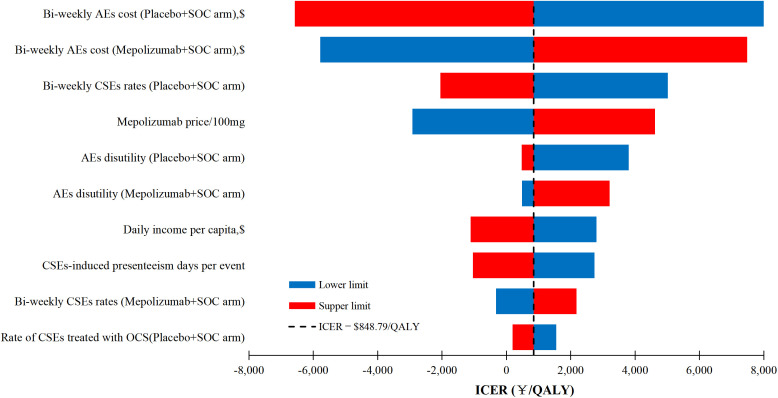
Deterministic sensitivity analyses result for mepolizumab versus placebo. ICER, incremental cost-effectiveness ratio; QALYs, quality-adjusted life-years; AEs, adverse events; SOC, standard of care; CSEs, clinically significant exacerbations; OCS, oral corticosteroid.

The DSA result depicted in Fig 2 highlight the substantial influence of bi-weekly AEs costs, bi-weekly CSEs rates, mepolizumab price, and AEs disutilities on the ICERs of mepolizumab treatment versus placebo. Remarkably, despite significant variations in all model parameters, the ICER between mepolizumab and placebo never surpassed the lower limit of the predefined WTP threshold range $15,217 per QALY). Detailed DSA results are further illustrated in S9 Table.

The PSA outcome was illustrated via a cost-effectiveness acceptability curve in S1 Fig, revealing that the probability of mepolizumab being considered cost-effectivenesss rises with higher WTP thresholds. Within the established WTP range of $15,217 to $38,042 per QALY, the likelihood of mepolizumab’s cost-effectiveness varied from 84% to 94%. Notably, at or above a WTP threshold of $22,706, the probability of mepolizumab being cost-effective consistently exceeded 90%. Additionally, the PSA result was visually depicted as a scatterplot in S2 Fig.

## Discussion

### Principal findings

This comprehensive economic evaluation was undertaken to systematically assess the cost-effectiveness of mepolizumab in the management of severe asthma among Chinese patients. The objective was to provide critical insights to inform clinical decision-making for this patient population. The principal findings are delineated as follows:

- The base-case analysis revealed that mepolizumab treatment not only incurred higher expenses but also enhanced survival when compared to placebo. Importantly, the ICER for mepolizumab relative to placebo was markedly below the lower bound of the predefined WTP threshold ($15,217 ~ $38,042 per QALY). This observation lends support to the implementation of mepolizumab among Chinese patients suffering from severe asthma.In our model, we separately considered drug costs, bi-weekly monitoring costs, CSEs management costs, productivity loss, and AEs management costs to explore the differences in their effects on offsetting the drug price (S10 Table). We found that the mepolizumab arm incurred lower costs for CSEs management, productivity loss, and AEs management compared to the placebo arm. Specifically, CSEs management costs were reduced by $44.99, productivity loss was reduced by $4,599.60, and AEs management costs were reduced by $1,868.90.
- Subgroup analyses, as detailed in S8 Table, have suggested that mepolizumab is the dominant treatment option within certain patient subgroups. Mepolizumab’s dominance in these subgroups is characterized by its cost-saving and life-extending effects. The pivotal factor behind its dominance is the lower risk of CSEs in the mepolizumab arm, which leads to lower costs associated with managing CSEs, reflected in reduced hospitalization and adverse event treatment costs. For example, the cost of Bi-weekly AEs in the mepolizumab group is $33.54, compared to $39.56 in the placebo group. This also results in a reduction in productivity loss due to the treatment of CSEs. Consequently, the total cost for patients receiving mepolizumab is actually lower than that for placebo. This finding has important implications for decision-making, suggesting that policymakers should give preferential consideration to mepolizumab for these specific patient profiles. Prioritizing mepolizumab in these cases could lead to optimized clinical outcomes and more efficient use of healthcare resources. Additionally, in other subgroups, the ICER for mepolizumab consistently remained below the lower threshold of the established WTP range, signifying its cost-effectiveness. These results bolster the rationale for employing mepolizumab across a spectrum of patient subgroups.
- The findings from DSA confirmed that, even under significant variations in model parameters, the ICER for mepolizumab versus placebo never exceeded the lower limit of the predefined WTP threshold range ($15,217 per QALY), thereby reinforcing the findings from the base-case and subgroup analyses. Although mepolizumab price ranked as the fourth most impactful model parameter, the drug has undergone price negotiations organized by China’s National Healthcare Security Administration-a policy-driven initiative with price reduction potential. Further price declines would enhance mepolizumab’s cost-effectiveness profile and could even yield cost-saving outcomes without compromising clinical efficacy. Additionally, PSA demonstrated a high probability (84% and 94%) of mepolizumab being cost-effective within the predefined WTP threshold range ($15,217 ~ $38,042) per QALY), underscoring its superior cost-effectiveness over placebo.

### Literature comparison analysis

A single existing study by Chaogang X et al. has previously assessed the cost-effectiveness of mepolizumab for the treatment of severe eosinophilic asthma in China, utilizing a Markov model and a lifetime treatment horizon [41]. Their findings estimated an ICER of $170,648.73 per QALY gained when mepolizumab is added to SOC, suggesting that it may not be cost-effective at its current price in China. This conclusion is in stark contrast to our ICER of $848.79 per QALY.

The discrepancy between our findings and those of Chaogang X et al. can be attributed to several key factors: ***Firstly***, our research was conducted from the Chinese society perspective, which enabled us to consider both direct medical costs and the substantial indirect costs related to productivity loss from CSEs. In contrast, Chaogang X et al. focused on the costs from the Chinese healthcare system perspective, potentially overlooking the broader economic impact. ***Secondly***, our study took a detailed approach to incorporating the impact of AEs on model outcomes, including their effects on health state utilities and the additional costs of treatment. This aspect was not included in the analysis by Chaogang X et al., which may have influenced their cost-effectiveness estimates. ***Thirdly***, In terms of mortality, our model applies state-specific mortality (No-CSEs vs. CSEs), which better reflects real-world mortality patterns and improves the accuracy of the cost-effectiveness analysis. By contrast, the previous study adopted a single, constant asthma-related mortality rate regardless of health state. This approach may underestimate mortality risk, particularly in high-risk populations, leading to an inflated ICER and a less favorable evaluation of mepolizumab’s cost-effectiveness.***Forthly,*** DSA in both studies shows that the price of mepolizumab and the discount rate significantly influence ICERs. In addition, our study further included AEs, and found that AE-related disutility and costs also substantially affected the ICER. PSA revealed that the probability of mepolizumab being cost-effective rose with increasing WTP, ranging from 84% to 94% at the predefined WTP threshold. By contrast, Chaogang X et al. reported that mepolizumab had zero probability of being cost-effective at the then-current price under a fixed WTP threshold..

### Strengths and limitation

This study boasts several significant strengths that enhance its importance. Firstly, this pioneering economic evaluation compares mepolizumab’s costs and outcomes for severe asthma in Chinese patients. Using a Chinese societal perspective and long-term horizon, the study assesses its effects on medical expenses, productivity, and quality of life, addressing a critical gap in the literature with insightful findings. Secondly, the study’s strength lies in its evaluation of cost-effectiveness across diverse subgroups, offering a nuanced understanding of how mepolizumab treatment affects various populations and demographic traits. This detailed analysis is essential for decision-makers, policymakers, and healthcare providers in optimizing resource allocation and treatment choices. Thirdly, the study meticulously incorporates the impact of AEs into the model, considering their implications on health state utilities and additional treatment costs, thereby ensuring a robust and reliable cost-effectiveness analysis.

This study also faces several limitations. ***Firstly***, the CSEs rate was derived from the MENSA trial, which may inherently introduce biases due to the trial’s specific design. Despite this, our sensitivity analysis has confirmed the stability of our results, with the ICER for mepolizumab consistently below the lower limit of the predefined WTP threshold range, even with substantial changes in CSEs rate-related parameters. ***Secondly,*** we have relied on health state utilities and cost parameters sourced from international literature, but the DSA has shown that potential variations in these parameters would not significantly impact the model’s ICER, implying that our conclusions would likely remain unchanged with more accurate utility and cost data (see Fig 2 and S9 Table for DSA results). ***Thirdly,*** the subgroup analysis should be interpreted with caution, given our assumption of a uniform CSEs rate across all subgroups in the placebo arm. Future studies incorporating subgroup-specific CSEs rate data would help optimize our findings. ***Fourthly,*** although our Markov model with a 14-day cycle length is grounded in empirical evidence, it may not fully capture the individual variability in exacerbation durations. ***Fifthly,*** our study failed to consider the potential impact of exacerbation history on the CSEs rate, a critical factor for predicting future exacerbations [42]. This oversight is due to the absence of direct empirical data in the Chinese context linking exacerbation history with CSE. Nonetheless, our sensitivity analysis has validated the consistency of our conclusions despite variations in CSEs rate-related parameters. ***Sixthly,*** we employed the human capital approach to quantify indirect costs attributable to asthma-related productivity loss, given its widespread use in cost-effectiveness research [43,44]. Although this method has inherent limitations, our deterministic sensitivity analysis demonstrated that fluctuations in key related parameters exerted minimal impact on the core outcomes of our model (see Fig 2 and S9 Table for DSA results). In summary, while our study is not without its limitations, the robustness of our findings, as evidenced by sensitivity analysis, indicates that our key conclusions are well-founded and reliable.

## Conclusion

From a Chinese societal perspective, the study concludes that mepolizumab is a cost-effective treatment option for severe asthma in China, applicable across various patient subgroups. This finding is crucial for healthcare decision-makers in China, where balancing cost and health outcomes is essential due to limited resources.

## Supporting information

S1 Table Dosage and administration for mepolizumab+SOC and placebo+SOC. (DOCX)

S2 Table Parameters for CSE rates. (DOCX)

S3 Table Data sources on asthma mortality rate calculation. (DOCX)

S4 Table Parameters for age-specific asthma mortality rates. (DOCX)

S5 Table Cost-related prarameters. (DOCX)

S6 Table Costs and disutilities for AE management. (DOCX)

S7 Table Parameters for health state utilities. (DOCX)

S8 Table Base-case and subgroup-level ICERs. (DOCX)

S9 Table DSA results in tabular format. (DOCX)

S10 Table Cost composition analysis of mepolizumab and placebo arms. (DOCX)

S1 Fig Cost-effectiveness acceptability curve for mepolizumab versus placebo. (DOCX)

S2 Fig Scatterplot for mepolizumab versus placebo. (DOCX)
