# Supplementary material for: Cost-effectiveness analysis of mepolizumab among patients with severe asthma from the Chinese societal perspective
Source: PLoS One. 2026 May 13;21(5):e0348955. doi: 10.1371/journal.pone.0348955 (PMC13170840; doi:10.1371/journal.pone.0348955)
Supplement: S1 Table — (DOCX) [file pone.0348955.s001.docx]

**S1 Table. Dosage and administration for placebo+SOC and mepolizumab+SOC ^[1]^**

| **Treatment** | **Dosage** | **Administration** |
| --- | --- | --- |
| Placebo+SOC | Prednisone: 10.6 mg | Daily |
| Mepolizumab+SOC | Mepolizumab: 100 mg | Every 4 weeks |
|  | Prednisone: 7.7 mg | Daily |

SOC, standard of care.

**Reference**

1.Chen R, Wei L, Dai Y, Wang Z, Yang D, Jin M, et al. Efficacy and safety of mepolizumab in a Chinese population with severe asthma: a phase III, randomised, double-blind, placebo-controlled trial. ERJ Open Res. 2024;10(3). Epub 20240520. doi: 10.1183/23120541.00750-2023. PubMed PMID: 38770009; PMCID: PMCPMC11103715.
