# Supplementary material for: Cost-effectiveness analysis of mepolizumab among patients with severe asthma from the Chinese societal perspective
Source: PLoS One. 2026 May 13;21(5):e0348955. doi: 10.1371/journal.pone.0348955 (PMC13170840; doi:10.1371/journal.pone.0348955)
Supplement: S2 Table — (DOCX) [file pone.0348955.s002.docx]

**S2 Table. Parameters for CSE rates**

| **Parameters** | **Baseline Value** | **Range for DSA** | **Distribution for PSA** | **Source** |
| --- | --- | --- | --- | --- |
| **Bi-weekly CSEs events** | | | | |
| Overall CSEs events rate(Placebo+SOC arm) | 0.05038 | 0.04031-0.06046 | Beta[α=91.13,β=1717.81] | MENSA study **^[1]^** |
| Overall CSEs events rate(Mepolizumab+SOC arm) | 0.01731 | 0.01385-0.02077 | Beta[α=94.38,β=5357.93] |  |
| Proportion of CSEs events treated with OCS(Placebo+SOC arm) | 0.89655 | 0.71724-1.00000 | Beta[α=15.09,β=1.74] |  |
| Proportion of CSEs events treated with OCS(Mepolizumab+SOC arm) | 0.89041 | 0.71233-1.00000 | Beta[α=15.24,β=1.88] |  |
| Proportion of CSEs events requiring hospitalisation and/or ED visit(Placebo+SOC arm) | 0.11330 | 0.09064-0.13596 | Beta[α=85.04,β=665.57] |  |
| Proportion of CSEs events requiring hospitalisation and/or ED visit(Mepolizumab+SOC arm) | 0.09589 | 0.07671-0.11507 | Beta[α=86.73,β=817.78] |  |
| Proportionof CSEs events requiring hospitalisation(Placebo+SOC arm) | 0.07389 | 0.05911-0.08867 | Beta[α=88.87,β=1113.81] |  |
| Proportion of CSEs events requiring hospitalisation(Mepolizumab+SOC arm) | 0.06849 | 0.05479-0.08219 | Beta[α=89.39,β=1215.70] |  |
| **RR in subgroups** | | | | |
| Age, years | | | | MENSA study **^[1]^** |
| <65 | 0.39 | 0.25-0.58 | Beta[α=15.45,β=24.17] |  |
| ≥65 | 0.21 | 0.07-0.62 | Beta[α=1.56,β=5.87] |  |
| Sex | | | |  |
| Male | 0.39 | 0.21-0.70 | Beta[α=5.55,β=8.68] |  |
| Female | 0.32 | 0.20-0.52 | Beta[α=10.13,β=21.52] |  |
| Weigh,kg | | | |  |
| <60 | 0.33 | 0.17-0.65 | Beta[α=4.32,β=9.19] |  |
| ＞60-≤75 | 0.22 | 0.12-0.39 | Beta[α=7.74,β=27.43] |  |
| ＞75 | 0.65 | 0.28-1.52 | Beta[α=0.83,β=0.45] |  |
| Baseline predicted pre-bronchodilator FEV1,% | | | |  |
| ≤60 | 0.29 | 0.18-0.48 | Beta[α=9.90,β=24.25] |  |
| ＞60-80 | 0.39 | 0.20-0.75 | Beta[α=4.32,β=6.76] |  |
| ＞80 | 0.53 | 0.12-2.31 | Beta[α=0.90,β=0.80] |  |
| Exacerbations in year prior to screening,n | | | |  |
| 2.00 | 0.33 | 0.18-0.58 | Beta[α=6.68,β=13.56] |  |
| 3.00 | 0.22 | 0.09-0.51 | Beta[α=3.07,β=10.88] |  |
| ≥4 | 0.45 | 0.25-0.81 | Beta[α=5.01,β=6.12] |  |
| Baseline maintenance OCS therapy | | | |  |
| Yes | 0.51 | 0.18-1.42 | Beta[α=0.76,β=0.73] |  |
| No | 0.33 | 0.22-0.49 | Beta[α=15.04,β=30.56] |  |
| Baseline airway reversibility | | | |  |
| Reversible | 0.41 | 0.24-0.69 | Beta[α=7.12,β=10.24] |  |
| Not reversible | 0.31 | 0.19-0.52 | Beta[α=9.05,β=20.14] |  |
| Blood eosinophil count at screening,cells/μL | | | |  |
| <150 | 0.20 | 0.08-0.53 | Beta[α=2.23,β=8.91] |  |
| ≥150-<300 | 0.38 | 0.17-0.83 | Beta[α=2.78,β=4.53] |  |
| 300-<500 | 0.50 | 0.20-1.21 | Beta[α=1.38,β=1.38] |  |
| ≥500 | 0.32 | 0.19-0.56 | Beta[α=7.50,β=15.93] |  |
| Randomization stratification factor:blood eosinophil count at screening,cells/μL | | | |  |
| ≥300 | 0.39 | 0.24-0.63 | Beta[α=8.98,β=14.05] |  |
| <300 | 0.32 | 0.17-0.59 | Beta[α=5.75,β=12.21] |  |

DSA, deterministic sensitivity analyses; PSA, probabilistic sensitivity analyses; CSEs, clinically significant exacerbations; SOC,standard of care; OCS, oral corticosteroid; ED:emergency department; FEV_1_, forced expiratory volume in one second.

**References**

1.Chen R, Wei L, Dai Y, Wang Z, Yang D, Jin M, et al. Efficacy and safety of mepolizumab in a Chinese population with severe asthma: a phase III, randomised, double-blind, placebo-controlled trial. ERJ Open Res. 2024;10(3). Epub 20240520. doi: 10.1183/23120541.00750-2023. PubMed PMID: 38770009; PMCID: PMCPMC11103715.
