# Supplementary material for: Cost-effectiveness analysis of mepolizumab among patients with severe asthma from the Chinese societal perspective
Source: PLoS One. 2026 May 13;21(5):e0348955. doi: 10.1371/journal.pone.0348955 (PMC13170840; doi:10.1371/journal.pone.0348955)
Supplement: S3 Table — (DOCX) [file pone.0348955.s003.docx]

**S3 Table. Data sources on asthma mortality rate calculation**

| **Age** | **Asthma deaths per 100,000^[1]^** | | **Asthma incidence Rate^[2]^** | | **Population^[3]^** | |
| --- | --- | --- | --- | --- | --- | --- |
|  | **Male** | **Female** | **Male** | **Female** | **Male** | **Female** |
| 50-54 | 0.78200 | 0.41200 | 4.10% | 4.50% | 61,105,470 | 60,058,826 |
| 55-59 | 1.24900 | 0.59600 | 4.10% | 4.50% | 50,816,026 | 50,584,760 |
| 60-64 | 2.02000 | 1.01700 | 6.40% | 5.60% | 36,871,125 | 36,511,813 |
| 65-69 | 3.81800 | 1.93000 | 6.40% | 5.60% | 36,337,923 | 37,667,637 |
| 70-74 | 8.56000 | 4.66700 | 8.50% | 6.50% | 24,162,733 | 25,427,303 |
| 75-79 | 16.79100 | 9.15800 | 8.50% | 6.50% | 14,752,433 | 16,486,416 |
| 80-84 | 32.10400 | 19.28700 | 8.50% | 6.50% | 9,157,003 | 11,225,875 |
| 85-89 | 69.27200 | 34.17100 | 8.50% | 6.50% | 4,426,091 | 6,400,439 |
| 90-94 | 110.24100 | 51.10200 | 8.50% | 6.50% | 1,367,594 | 2,285,155 |
| 95+ | 98.42000 | 74.90200 | 8.50% | 6.50% | 306,584 | 632,094 |

**References**

1.Yu C. Study on the status and trends of asthma death burden in China: Nanchang University; 2023.

2.Huang K, Yang T, Xu J, Yang L, Zhao J, Zhang X, et al. Prevalence, risk factors, and management of asthma in China: a national cross-sectional study. Lancet. 2019;394(10196):407-18. Epub 20190620. doi: 10.1016/s0140-6736(19)31147-x. PMID: 31230828.

3.National Bureau of Statistics. China population census yearbook 2020. Available from: <https://www.stats.gov.cn/sj/pcsj/rkpc/7rp/indexch.htm>.
