# Supplementary material for: Cost-effectiveness analysis of mepolizumab among patients with severe asthma from the Chinese societal perspective
Source: PLoS One. 2026 May 13;21(5):e0348955. doi: 10.1371/journal.pone.0348955 (PMC13170840; doi:10.1371/journal.pone.0348955)
Supplement: S4 Table — (DOCX) [file pone.0348955.s004.docx]

**S4 Table . Parameters for age-specific asthma mortality rates**

| **Age** | **Bi-weekly asthma mortality rate (No-CSEs population)** | **Bi-weekly asthma mortality rate**  **(CSEs population)** |
| --- | --- | --- |
| 50-54 | 0.000005445 | 0.000842003 |
| 55-59 | 0.000008414 | 0.000842003 |
| 60-64 | 0.000009576 | 0.001643305 |
| 65-69 | 0.000018017 | 0.001643305 |
| 70-74 | 0.000033046 | 0.002086932 |
| 75-79 | 0.000064531 | 0.002086932 |
| 80-84 | 0.000128321 | 0.003679127 |
| 85-89 | 0.000248448 | 0.003679127 |
| 90-94 | 0.000377708 | 0.007429295 |
| 95+ | 0.000446385 | 0.007429295 |

CSEs, clinically significant exacerbations
