# Supplementary material for: Cost-effectiveness analysis of mepolizumab among patients with severe asthma from the Chinese societal perspective
Source: PLoS One. 2026 May 13;21(5):e0348955. doi: 10.1371/journal.pone.0348955 (PMC13170840; doi:10.1371/journal.pone.0348955)
Supplement: S5 Table — (DOCX) [file pone.0348955.s005.docx]

**S5 Table. Cost-related prarameters**

| **Parameters** | **Baseline Value** | **Range for DSA** | **Distribution for PSA** | **Source** |
| --- | --- | --- | --- | --- |
| **Treatment costs** | | | | |
| Mepolizumab price/100mg,**$** | 682.02 | 545.62-818.43 | Gamma[α=25,λ=0.0052] | National Health Industry Data Platform**^[1]^** |
| Prednisone price/5mg,**$** | 0.89 | 0.71-1.07 | Gamma[α=24.84,λ=3.96] |  |
| **Monitoring and exacerbation costs** | | | | |
| Bi-weekly monitoring cost,**$** | 4.78 | 3.82-5.73 | Gamma[α=25.03,λ=0.74] | Zhou K, et al**^[2]^** |
| CSEs management costs | | | |  |
| —Event cost of CSEs requiring hospitalisation and/or ED visit,**$** | 254.91 | 203.93-305.89 | Gamma[α=25,λ=0.014] |  |
| —Event cost of CSEs requiring hospitalisation,**￥($)** | 353.80 | 283.04-424.56 | Gamma[α=25,λ=0.010] |  |
| —Daily cost of CSEs treated with OCS,**$** | 7.13 | 5.70-8.56 | Gamma[α=24.99,λ=0.50] | National Health Industry Data Platform**^[1]^** |
| —Days of CSEs treated with OCS (Placebo + SOC arm) | 10.10 | 2.10-18.10 | Gamma[α=25.00,λ=2.48] | MENSA study **^[3]^** |
| —Days of CSEs treated with OCS (Mepolizumab + SOC arm) | 8.10 | 2.20-14.00 | Gamma[α=25.00,λ=3.09] |  |
| **Productivity loss** | | | | |
| Daily income per capita,**$** | 15.25 | 12.20-18.30 | Gamma[α=25.00,λ=0.23] | National Bureau of Statistics **^[4]^** |
| Employment rate (%) | | | |  |
| —50-54 years old | 67.12% | 53.70%-80.55% | Beta |  |
| —55-59 years old | 54.19% | 43.35%-65.03% | Beta |  |
| —60-64 years old | 33.84% | 27.07%-40.61% | Beta |  |
| —65-69 years old | 26.96% | 21.57%-32.35% | Beta |  |
| —70-74 years old | 17.37% | 13.89%-20.84% | Beta |  |
| —75+ years old | 6.53% | 5.23%-7.84% | Beta |  |
| Presenteeism days due to CSEs per event | 62.00 | 49.60-74.40 | Gamma[α=25.00,λ=0.40] | Finkelstein EA，et al **^[5]^** |
| Days of workloss due to CSEs per event | | | | |
| —CSEs requiring hospitalisation and/or ED visit | 6.40 | 5.12-7.68 | Gamma[α=25.00,λ=3.91] | Lin HC, et al**^[6]^** |
| —CSEs requiring hospitalisation | 6.40 | 5.12-7.68 | Gamma[α=25.00,λ=3.91] |  |
| —CSEs treated with OCS | 1.00 | 0.80-1.20 | Gamma[α=25.00,λ=25.00] |  |
| **AEs management** | | | | |
| Bi-weekly AEs cost (Placebo+SOC arm),**$** | 39.56 | 31.65-47.48 | Gamma[α=25.00,λ=0.09] | Estimated (Table S6) |
| Bi-weekly AEs cost (Mepolizumab+SOC arm),**$** | 33.54 | 26.83-40.25 | Gamma[α=25.00,λ=0.11] |  |

DSA, deterministic sensitivity analyses; PSA, probabilistic sensitivity analyses; CSEs, clinically significant exacerbations; ED:emergency department; OCS, oral corticosteroid; SOC,standard of care; AEs, adverse events.

**References**

1.National Bureau of Statistics. Annual data. Available from: <https://data.stats.gov.cn/easyquery.htm?cn=C01>

2.Zhou K, Zhang M, Zuo C, Xie X, Xuan J. Cost-effectiveness analysis of budesonide/formoterol SMART therapy versus salmeterol/fluticasone plus as-needed SABA among patients ≥12 years with moderate asthma from the Chinese societal perspective. J Med Econ. 2024;27(1):1018-26. Epub 20240817. doi: 10.1080/13696998.2024.2385191. PMID: 39067014.

3.Chen R, Wei L, Dai Y, Wang Z, Yang D, Jin M, et al. Efficacy and safety of mepolizumab in a Chinese population with severe asthma: a phase III, randomised, double-blind, placebo-controlled trial. ERJ Open Res. 2024;10(3). Epub 20240520. doi: 10.1183/23120541.00750-2023. PubMed PMID: 38770009; PMCID: PMCPMC11103715.

4.National Bureau of Statistics. China population census yearbook 2020. Available from: <https://www.stats.gov.cn/sj/pcsj/rkpc/7rp/indexch.htm>.

5. Finkelstein EA, Lau E, Doble B, Ong B, Koh MS. Economic burden of asthma in Singapore. BMJ Open Respir Res. 2021;8(1). doi: 10.1136/bmjresp-2020-000654. PubMed PMID: 33737309; PMCID: PMCPMC7978329.

6. Lin HC, Kao S, Wen HC, Wu CS, Chung CL. Length of stay and costs for asthma patients by hospital characteristics--a five-year population-based analysis. J Asthma. 2005;42(7):537-42. doi: 10.1080/02770900500214783. PMID: 16169785.
