# Supplementary material for: Cost-effectiveness analysis of mepolizumab among patients with severe asthma from the Chinese societal perspective
Source: PLoS One. 2026 May 13;21(5):e0348955. doi: 10.1371/journal.pone.0348955 (PMC13170840; doi:10.1371/journal.pone.0348955)
Supplement: S6 Table — (DOCX) [file pone.0348955.s006.docx]

**S6 Table. Costs and disutilities for AE management**

| **AEs** | **Disutilities^[1]^** | **Cost per eventa,$** |
| --- | --- | --- |
| Asthma | 0.05000 | 630.65 |
| Upper respiratory tract infection | 0.04600 | 484.62 |
| Nasopharyngitis | 0.07346 | 437.08 |
| Bronchitis | 0.07346 | 630.65 |
| Rhinitis allergic | 0.07346 | 437.08 |
| Productive cough | 0.04600 | 772.28 |
| Pneumonia | 0.07346 | 484.62 |
| Headache | 0.06900 | 484.62 |
| Cough | 0.04600 | 772.28 |
| Dizziness | 0.07346 | 321.85 |
| Pharyngitis | 0.07346 | 392.95 |
| Rhinitis | 0.07346 | 437.08 |
| Arthralgia | 0.06900 | 609.93 |
| Oropharyngeal pain | 0.06900 | 437.08 |

AEs, adverse event.

^a^The cost of AEs per event is determined based on China's Diagnosis-Related Groups policy, which establishes a payment standard for diseases classified according to the Diagnosis Classification and Codes (ICD-10). For each AE in our model, the corresponding industry average medical fees are determined via the ICD-10 directory, ensuring a standardized approach to cost inclusion.

**References**

1.Lin HC, Kao S, Wen HC, Wu CS, Chung CL. Length of stay and costs for asthma patients by hospital characteristics--a five-year population-based analysis. J Asthma. 2005;42(7):537-42. doi: 10.1080/02770900500214783. PMID: 16169785.
