# Supplementary material for: Cost-effectiveness analysis of mepolizumab among patients with severe asthma from the Chinese societal perspective
Source: PLoS One. 2026 May 13;21(5):e0348955. doi: 10.1371/journal.pone.0348955 (PMC13170840; doi:10.1371/journal.pone.0348955)
Supplement: S7 Table — (DOCX) [file pone.0348955.s007.docx]

**S7 Table. Parameters for health state utilities**

| **Parameters** | **Baseline Value** | **Range for DSA** | **Distribution for PSA** | **Source** |
| --- | --- | --- | --- | --- |
| No exacerbations | 0.84000 | 0.83000-0.86000 | Beta[α=1926.72.00,β=367.00] | Oh BC, et al**^[1]^** |
| **Disutility of exacerbation** | | | | |
| —CSEs treated with OCS | 0.10000 | 0.08000-0.12000 | Beta[α=86.34,β=777.02] | Zhou K, et all**^[2]^** |
| —CSEs requiring hospitalisation and/or ED visit | 0.15000 | 0.12000-0.18000 | Beta[α=81.48,β=461.74] |  |
| —CSEs requiring hospitalisation | 0.20000 | 0.16000-0.24000 | Beta[α=76.63,β=306.53] |  |
| **AEs disutility** | | | | |
| AEs disutility (Placebo+SOC arm) | 0.10763 | 0.08610-0.12915 | Beta[α=85.60,β=709.73] | Estimated (Table S6) |
| AEs disutility (Mepolizumab+SOC arm) | 0.09666 | 0.07733-0.11599 | Beta[α=86.65,β=809.88] |  |

DSA, deterministic sensitivity analyses; PSA, probabilistic sensitivity analyses; CSEs, clinically significant exacerbations; OCS, oral corticosteroid; ED, emergency department; SOC, standard of care.

**References**

1.Oh BC, Lee JE, Nam JH, Hong JY, Kwon SH, Lee EK. Health-related quality of life in adult patients with asthma according to asthma control and severity: A systematic review and meta-analysis. Front Pharmacol. 2022;13:908837. Epub 20221121. doi: 10.3389/fphar.2022.908837. PubMed PMID: 36479200; PMCID: PMCPMC9720394.

2.Zhou K, Zhang M, Zuo C, Xie X, Xuan J. Cost-effectiveness analysis of budesonide/formoterol SMART therapy versus salmeterol/fluticasone plus as-needed SABA among patients ≥12 years with moderate asthma from the Chinese societal perspective. J Med Econ. 2024;27(1):1018-26. Epub 20240817. doi: 10.1080/13696998.2024.2385191. PMID: 39067014.
