# Supplementary material for: Cost-effectiveness analysis of mepolizumab among patients with severe asthma from the Chinese societal perspective
Source: PLoS One. 2026 May 13;21(5):e0348955. doi: 10.1371/journal.pone.0348955 (PMC13170840; doi:10.1371/journal.pone.0348955)
Supplement: S8 Table — (DOCX) [file pone.0348955.s008.docx]

**S8 Table. Base-case and subgroup-level ICERs**

| **Treatment regimen** | | **Cost,$** | **QALYs** | **Incremental costs,$** | **Incremental QALYs** | **ICER,$**/QALY |
| --- | --- | --- | --- | --- | --- | --- |
| **Total patients population** | | | | | | |
| Placebo+SOC | | 30,302.61 | 13.04657 | NA | NA | NA |
| Mepolizumab+SOC | | 30,701.78 | 13.51685 | 399.17 | 0.47028 | 848.79 |
| **Subgroups** | | | | | | |
| Age, years | | | | | | |
| <65 | Mepolizumab+SOC | 31,078.79 | 13.49594 | 776.18 | 0.44937 | 1,727.25 |
| ≥65 |  | 29,604.03 | 13.57801 | -698.58 | 0.53144 | Dominance |
| Sex |  |  |  |  |  |  |
| Male | Mepolizumab+SOC | 31,078.79 | 13.49594 | 776.18 | 0.44937 | 1,727.25 |
| Female |  | 30,509.31 | 13.52754 | 206.69 | 0.48097 | 429.74 |
| Weigh, kg | | | | | | |
| <60 | Mepolizumab+SOC | 30,590.97 | 13.52300 | 288.36 | 0.47643 | 605.25 |
| ＞60-≤75 |  | 29,686.86 | 13.57338 | -615.75 | 0.52681 | Dominance |
| ＞75 |  | 33,150.42 | 13.38197 | 2,847.81 | 0.33540 | 8,490.85 |
| Baseline predicted pre-bronchodilator FEV1, % | | | | | | |
| ≤60 | Mepolizumab+SOC | 33,150.42 | 13.54121 | -38.93 | 0.49464 | Dominance |
| ＞60-80 |  | 31,078.79 | 13.49594 | 776.18 | 0.44937 | 1,727.25 |
| ＞80 |  | 32,202.71 | 13.43392 | 1,900.10 | 0.38735 | 4,905.36 |
| Exacerbations in year prior to screening, n | | | | | | |
| 2.00 | Mepolizumab+SOC | 30,590.97 | 13.52300 | 288.36 | 0.47643 | 605.25 |
| 3.00 |  | 29,686.86 | 13.57338 | -615.75 | 0.52681 | Dominance |
| ≥4 |  | 31,562.91 | 13.46917 | 1,260.30 | 0.42260 | 2,982.22 |
| Baseline maintenance OCS therapy | | | | | | |
| Yes | Mepolizumab+SOC | 31,562.91 | 13.44269 | 1,740.75 | 0.39612 | 4,394.53 |
| No |  | 30,590.97 | 13.52300 | 288.36 | 0.47643 | 605.25 |
| Baseline airway reversibility | | | | | | |
| Reversible | Mepolizumab+SOC | 31,240.58 | 13.48699 | 937.96 | 0.44042 | 2,129.71 |
| Not reversible |  | 30,427.53 | 13.53209 | 124.92 | 0.48552 | 257.30 |
| Blood eosinophil count at screening, cells/μL | | | | | | |
| <150 | Mepolizumab+SOC | 30,427.53 | 13.58265 | -781.52 | 0.53608 | Dominance |
| ≥150-<300 |  | 30,997.75 | 13.50043 | 695.14 | 0.45386 | 1,531.60 |
| 300-<500 |  | 31,963.54 | 13.44708 | 1,660.93 | 0.40051 | 4,147.01 |
| ≥500 |  | 30,509.31 | 13.52754 | 206.69 | 0.48097 | 429.74 |
| Randomization stratification factor:blood eosinophil count at screening, cells/μL | | | | | | |
| ≥300 | Mepolizumab+SOC | 30,701.78 | 13.51685 | 399.17 | 0.47028 | 848.79 |
| <300 |  | 30,509.31 | 13.52754 | 206.69 | 0.48097 | 429.74 |

ICER, incremental cost-effectiveness ratio; QALYs, quality-adjusted life-years; SOC, standard of care; FEV_1_, forced expiratory volume in one second; OCS, oral corticosteroid.
