# Supplementary material for: Cost-effectiveness analysis of mepolizumab among patients with severe asthma from the Chinese societal perspective
Source: PLoS One. 2026 May 13;21(5):e0348955. doi: 10.1371/journal.pone.0348955 (PMC13170840; doi:10.1371/journal.pone.0348955)
Supplement: S9 Table — (DOCX) [file pone.0348955.s009.docx]

**S9 Table. DSA results in tabular format**

| **Parameters** | **Impact on ICER** | **Generated low ICER value, ($/QALY)** | **Generated high ICER value, ($/QALY)** | **Spread, ($/QALY)** |
| --- | --- | --- | --- | --- |
| Bi-weekly AEs cost (Placebo+SOC arm) | Decrease | -6,577.73 | 8,275.31 | 14,853.04 |
| Bi-weekly AEs cost (Mepolizumab+SOC arm) | Increase | -5,782.92 | 7,480.51 | 13,263.43 |
| Mepolizumab price/100mg | Increase | 13,263.43 | 4,619.43 | 7,541.29 |
| Bi-weekly CSEs rate(Placebo+SOC arm) | Decrease | -2,050.14 | 5,015.69 | 7,065.83 |
| Daily income per capita | Decrease | -1,107.30 | 2,804.89 | 3,912.19 |
| Presenteeism days due to CSEs per event | Decrease | -1,040.88 | 2,738.47 | 3,779.35 |
| AEs disutility (Placebo+SOC arm) | Decrease | 477.65 | 3,806.77 | 3,329.13 |
| AEs disutility (Mepolizumab+SOC arm) | Increase | 489.18 | 3,204.60 | 2,715.42 |
| Bi-weekly CSEs rate (Mepolizumab+SOC arm) | Increase | -325.32 | 2,177.11 | 2,502.42 |
| Rate of CSEs treated with OCS(Placebo+SOC arm) | Decrease | 190.11 | 1,551.84 | 1,361.72 |
| Days of CSEs treated with OCS (Placebo + SOC arm) | Decrease | 238.84 | 1,458.74 | 1,219.90 |
| Daily cost of CSEs treated with OCS | Decrease | 414.67 | 1,282.91 | 868.24 |
| Rate of CSEs requiring hospitalisation and/or ED visit(Placebo+SOC arm) | Decrease | 538.20 | 1,282.91 | 625.04 |
| Rate of CSEs requiring hospitalisation(Placebo+SOC arm) | Decrease | 576.27 | 1,124.25 | 547.98 |
| Rate of CSEs treated with OCS(Mepolizumab+SOC arm) | Increase | 650.24 | 1,052.05 | 401.81 |
| Cost of CSEs requiring hospitalisation and/or ED visit per event | Decrease | 659.51 | 1,038.07 | 378.56 |
| Days of CSEs treated with OCS (Mepolizumab + SOC arm) | Increase | 672.96 | 1,024.62 | 351.66 |
| Cost of CSEs requiring hospitalisation per event | Decrease | 684.66 | 1,012.92 | 328.26 |
| Rate of CSEs requiring hospitalisation and/or ED visit(Mepolizumab+SOC arm) | Increase | 753.37 | 944.57 | 191.20 |
| Rate of CSEs requiring hospitalisation(Mepolizumab+SOC arm) | Increase | 757.13 | 940.78 | 183.65 |
| Prednisone price/5mg | Decrease | 767.20 | 930.38 | 163.18 |
| Days of workloss due to CSEs(requiring hospitalisation) | Decrease | 809.80 | 887.78 | 77.98 |
| Days of workloss due to CSEs(requiring hospitalisation and/or ED visit) | Decrease | 809.80 | 887.78 | 77.98 |
| Days of workloss due to CSEs(treated with OCS) | Decrease | 821.36 | 876.22 | 54.86 |
| Cost of bi-weekly monitoring | Increase | 829.66 | 867.93 | 38.27 |
| Disutility of CSEs(treated with OCS) | Decrease | 831.44 | 866.88 | 35.44 |
| Utility of No-CSEs health state | Decrease | 835.92 | 855.38 | 19.46 |
| Disutility of CSEs(requiring hospitalisation and/or ED visit) | Decrease | 845.17 | 852.44 | 7.27 |
| Disutility of CSEs(requiring hospitalisation) | Decrease | 845.77 | 851.83 | 6.06 |

DSA, deterministic sensitivity analyses; ICER, incremental cost-effectiveness ratio; QALYs, quality-adjusted life-years; AEs, adverse events; SOC,standard of care; CSEs, clinically significant exacerbations; OCS, oral corticosteroid; ED:emergency department.
