# Supplementary material for: Cost-effectiveness analysis of mepolizumab among patients with severe asthma from the Chinese societal perspective
Source: PLoS One. 2026 May 13;21(5):e0348955. doi: 10.1371/journal.pone.0348955 (PMC13170840; doi:10.1371/journal.pone.0348955)
Supplement: S10 Table — (DOCX) [file pone.0348955.s010.docx]

**S10 Table. Cost Composition Analysis of Mepolizumab and Placebo arms**

| **Treatment regimen** | | **Cost,$** | **Incremental costs,$** |
| --- | --- | --- | --- |
| **Total patients population** | | | |
| Placebo+SOC | | 30,302.61 | NA |
| Mepolizumab+SOC | | 30,701.78 | 399.17 |
| drug cost | Placebo+SOC | 817.22 | NA |
|  | Mepolizumab+SOC | 9,491.65 | 8,674.42 |
| Bi-weekly monitoring cost | Placebo+SOC | 2,215.08 | NA |
|  | Mepolizumab+SOC | 2,260.07 | 44.99 |
| CSEs management costs | Placebo+SOC | 265.60 | NA |
|  | Mepolizumab+SOC | 804.81 | -1,851.79 |
| Productivity loss | Placebo+SOC | 7,151.01 | NA |
|  | Mepolizumab+SOC | 2,551.46 | -4,599.60 |
| AEs management cost | Placebo+SOC | 17,462.70 | NA |
|  | Mepolizumab+SOC | 15,593.80 | -1,868.90 |
