# Supplementary material for: Cost-effectiveness analysis of mepolizumab among patients with severe asthma from the Chinese societal perspective
Source: PLoS One. 2026 May 13;21(5):e0348955. doi: 10.1371/journal.pone.0348955 (PMC13170840; doi:10.1371/journal.pone.0348955)
Supplement: S1 Fig — (DOCX) [file pone.0348955.s011.docx]

**S1 Fig. Cost-effectiveness acceptability curve for mepolizumab versus placebo**

**
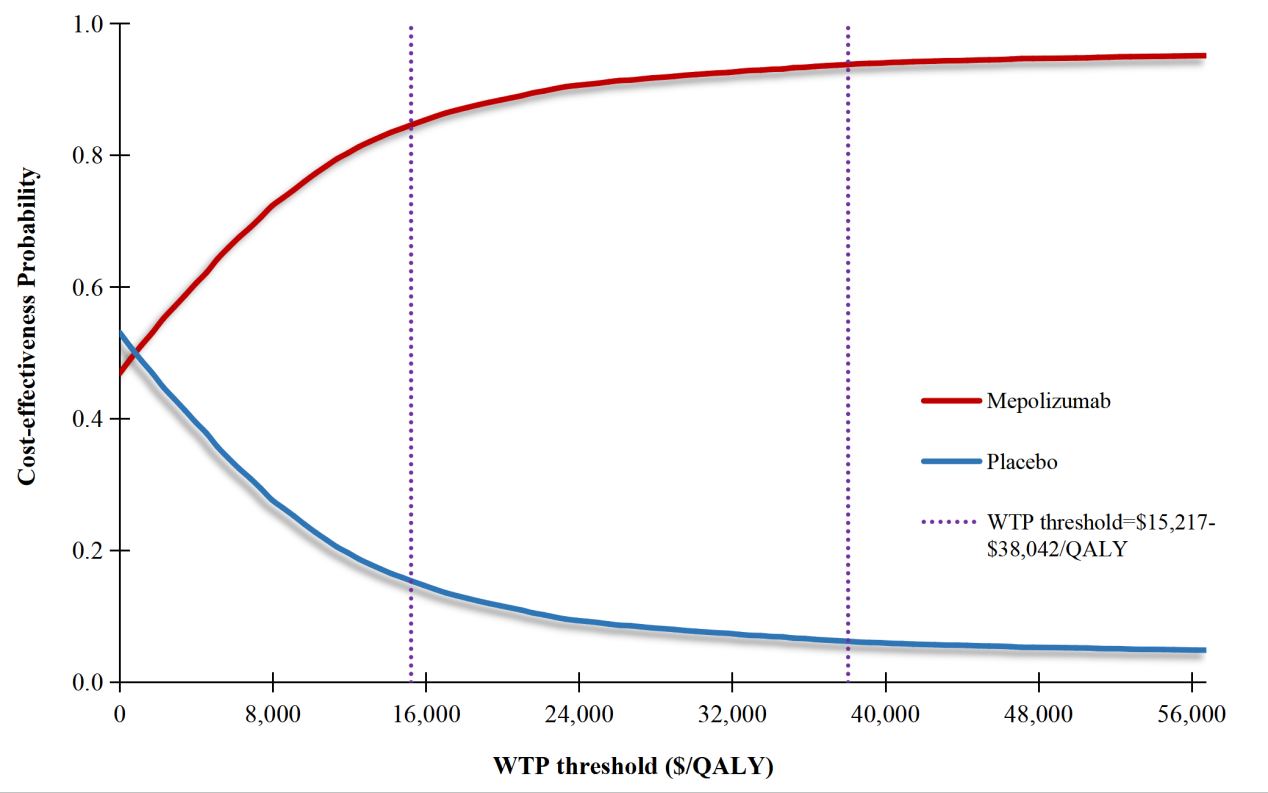
**

WTP, willingness-to-pay; QALY, quality-adjusted life-year.
