# Supplementary figures and images for: Cost-effectiveness analysis of mepolizumab among patients with severe asthma from the Chinese societal perspective
Source: PLoS One. 2026 May 13;21(5):e0348955. doi: 10.1371/journal.pone.0348955 (PMC13170840; doi:10.1371/journal.pone.0348955)

**S2 Fig. Scatterplot for mepolizumab versus placebo**

**
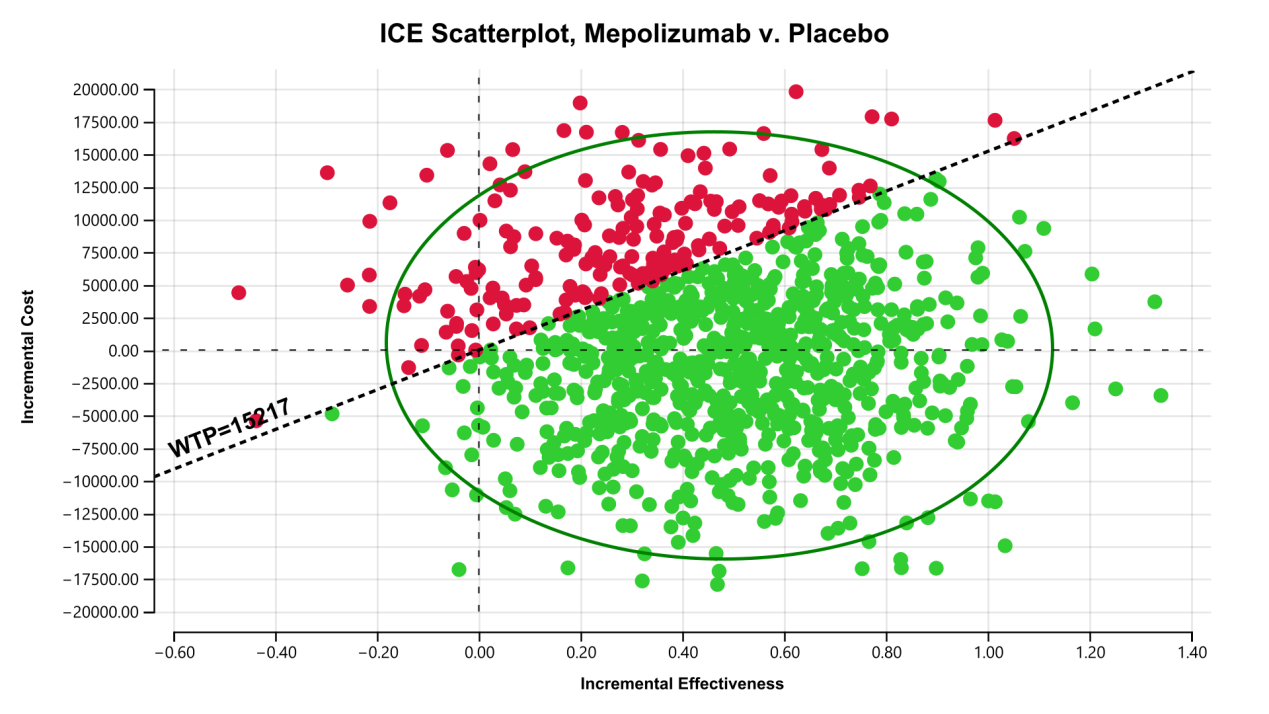
**

ICE, incremental effectiveness.

Supplement: S2 Fig — (DOCX) [file pone.0348955.s012.docx]
